# HDAC-4 regulates claudin-2 expression in EGFR-ERK1/2 dependent manner to regulate colonic epithelial cell differentiation

**DOI:** 10.18632/oncotarget.21190

**Published:** 2017-09-23

**Authors:** Rizwan Ahmad, Balawant Kumar, Kaichao Pan, Punita Dhawan, Amar B. Singh

**Affiliations:** ^1^ Department of Biochemistry and Molecular Biology, University of Nebraska Medical Center, Omaha, NE, USA; ^2^ VA Nebraska-Western Iowa Health Care System, Omaha, NE, USA

**Keywords:** epigenetic regulation, differentiation, colon cancer, cell cycle, claudin

## Abstract

In normal colon, claudin-2 expression is restricted to the crypt bottom containing the undifferentiated and proliferative colonocytes. Claudin-2 expression is also upregulated in colorectal cancer (CRC) and promotes carcinogenesis. However, cellular mechanism/s regulated by increased claudin-2 expression during the CRC and mechanism/s regulating this increase remain poorly understood. Epigenetic mechanisms help regulate expression of cancer-associated genes and inhibition of Histone Deacetylases (HDACs) induces cell cycle arrest and differentiation. Accordingly, based on a comprehensive *in vitro* and *in vivo* analysis we here report that Histone Deacetylases regulate claudin-2 expression in causal association with colonocyte dedifferentiation to promote CRC. Detailed differentiation analyses using colon cancer cells demonstrated inverse association between claudin-2 expression and epithelial differentiation. Genetic manipulation studies revealed the causal role of HDAC-4 in regulating claudin-2 expression during this process. Further analysis identified transcriptional regulation as the underlying mechanism, which was dependent on HDAC-4 dependent modulation of the EGFR-ERK1/2 signaling. Accordingly, colon tumors demonstrated marked upregulation of the HDAC-4/ERK1/2/Claudin-2 signaling. Taken together, we demonstrate a novel role for HDAC-4/EGFR/ERK1/2 signaling in regulating claudin-2 expression to modulate colonocyte differentiation. These findings are of clinical significance and highlight epigenetic regulation as potential mechanism to regulate claudin-2 expression during mucosal pathologies including CRC.

## INTRODUCTION

The colonic epithelial barrier is a dynamic system renewed continuously by the process of colonocyte proliferation and migration from the proliferative crypt bottom. Epithelial cells creating this barrier are tethered together by intercellular junctional complexes where tight junction (TJ), the most apical cell-cell adhesion, helps create a regulated barrier to the paracellular transport [1, 2]. Studies have now demonstrated that the TJ-integral and associated proteins also help regulate the processes of proliferation, differentiation, and apicobasal polarity [3–6]. Accordingly, tight junctions are subject to dynamic modulation during epithelial tissue remodeling, wound repair, inflammation, and tumorigenesis [6–10]. The claudin family of proteins is integral constituent of the tight junction and expressed in tissue specific manner [9]. Thus, specific claudin subtype combination can differentiate properties of epithelial cells in terms of their physiological and pathological functions. In this regard, claudin-2 is unique among claudin proteins as its expression is restricted primarily to the leaky epithelia. Accordingly, in the colon, claudin-2 expression is particularly abundant in proliferative and undifferentiated colonocytes at the crypt base, which is also leakier than the crypt top [3, 10, 11].

Dedifferentiation of epithelial cells and unchecked proliferation helps promote malignant growth including CRC. Our laboratory, and of others, have demonstrated that claudin-2 expression is highly upregulated in CRC. Moreover, forced claudin-2 expression in claudin-2 deficient CRC cells promoted *in-vivo* tumor growth [3]. Similar upregulation of claudin-2 expression is now reported in lung, liver, stomach cancer tissues and to promote breast cancer metastasis [3, 12–15]. Dedifferentiation promotes tumorigenic and metastatic abilities of cancer cells [16–18]. However, despite evidences suggesting an association between claudin-2 expression and colonic epithelial differentiation, a causal association, and underlying regulatory mechanisms remain poorly understood.

Recent studies have highlighted importance of the epigenetic mechanisms such as histone modifications, DNA methylation and chromatin remodeling in the pathobiology of CRC [19–21]. Among them, histone deacetylase (HDAC)-mediated epigenetic regulation plays central role in the homoeostasis of histone acetylation, gene transcription and therefore regulation of specific genes implicated in growth arrest, terminal differentiation and apoptosis [22, 23]. Previous studies from our laboratory, and of others, have highlighted epigenetic regulation as potential mechanism controlling deregulation of claudin proteins in cancer cells and tissues [24–27]. Moreover, several inhibitors of the HDACs have been developed and approved by the FDA for testing their therapeutic efficacy in limiting solid tumors and hematological malignancies [28–30]. It is here worthy of noting that the conventional anti-cancer strategies have shown limited success in clinical management of the disease. Thus, finding better therapeutics targets to prevent CRC and associated patient death remains a priority.

In present study, we report a key role of claudin-2 expression in regulating differentiation among colonocytes and colon cancer cells as claudin-2 expression antagonized epithelial differentiation. We therefore hypothesized that reduction of claudin-2 expression could reduce the CRC tumor burden. In support, we provide evidence that claudin-2 expression in CRC is epigenetically regulated in manners dependent on HDAC4/EGFR/ERK1/2 signaling, key signaling mechanisms implicated in CRC growth and progression [3]. Our findings highlight therapeutic significance of the HDACi in inhibiting the EGFR-ERK1/2-Claudin-2 signaling for treating high claudin-2 expressing CRC patients.

## RESULTS

### Claudin-2 expression decreases with colonocyte differentiation

**Figure 1:**
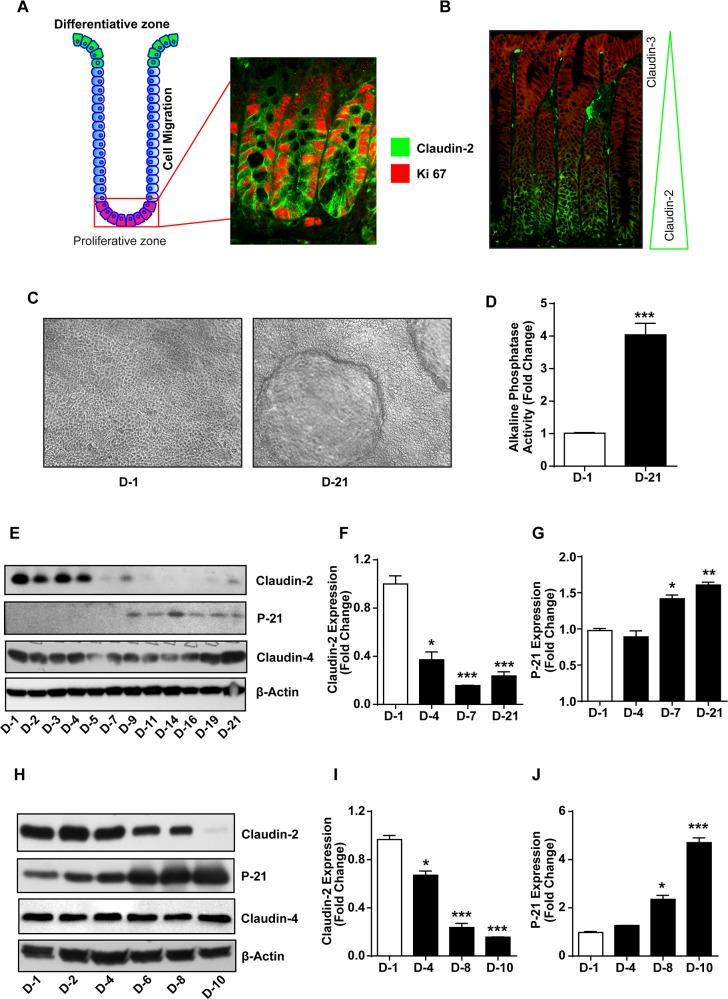
Colonic claudin-2 expression is restricted to proliferative crypt base and decreases with colonic epithelial differentiation **(A)** Cartoon depicting normal organization of a colonic crypt and differentiation zone, and co-immunoflourescent localization using anti-claudin-2 (green) and Ki-67 (red) antibodies.; **(B)** Immunofluorescence staining using anti-claudin-2 (green) and claudin-3 (red) antibodies showing distinct and specific pattern of claudin expression in the colonic crypt.; **(C-D)** Caco-2 cells make dome like structures and demonstrate increased alkaline phosphatase (AP) activity as they undergo spontaneous differentiation.; **(E-J)** Immunoblot with representative densitometry analysis using total cell lysate from Caco-2 and HT29 cells subjected to spontaneous differentiation, representing claudin-2 claudin-4 and P-21^waf1/cip1^Immunoblot. Three independent experiments were done and data is presented as mean ± S.E.M. ^*^P<0.05, ^**^P<0.01 and ^***^ P<0.001 *versus* control.

**Figure 2:**
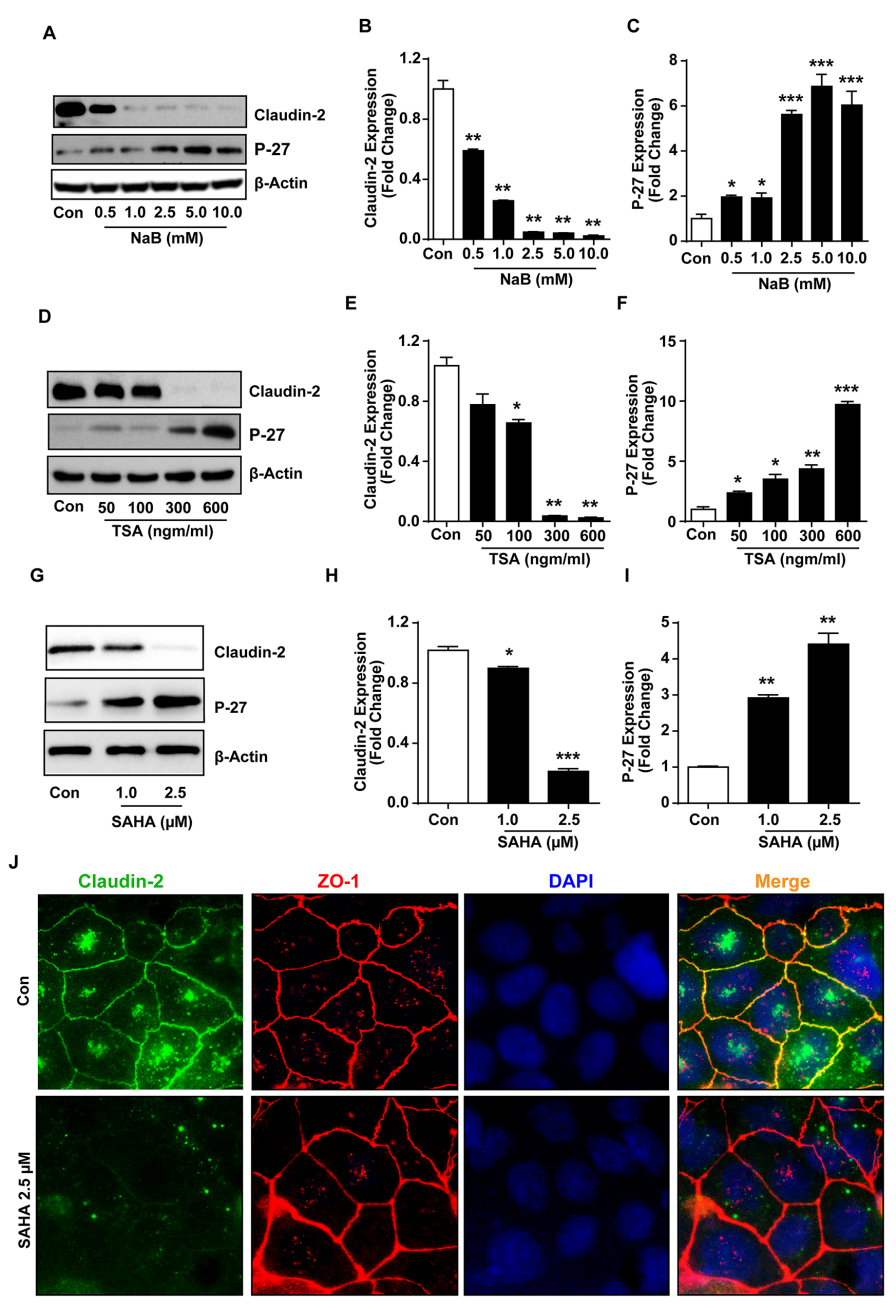
Histone deacetylase (HDACs) inhibitors (HDACi) induce differentiation and suppress claudin-2 expression in colon cancer cells **(A-C)** Immunoblot and densitometry analysis of claudin-2 and P-27^Kip1^expression in Caco-2 cell treated with sodium butyrate (NaB).; **(D-F)** Trichostatin A (TSA) treated Caco-2 cell representing Immunoblot and densitometry of claudin-2 and P-27^Kip1^.; **(G-I)** Representative immunoblot and densitometry of claudin-2 and P27^Kip1^ in suberoylanilide hydroxamic acid (SAHA) treated Caco-2 cell lysate.; **(J)** Representative images from the immunofloresence analysis for claudin-2 expression and cellular distribution in Caco-2 cells subjected to SAHA-treatment. Co-localization with Zo-1 served as control. At least three independent experiments were done and data is presented as mean ± S.E.M. ^*^P<0.05, ^**^P<0.01 and ^***^ P<0.001 *versus* control.

As described, colonic claudin-2 expression is concentrated among undifferentiated and proliferative colonocytes at the crypt bottom. Co-immunofluorescent localization of claudin-2 and Ki67, a proliferative marker, supported this assertion. Specificity of this peculiar tissue distribution was further supported by the co-immunofluorescent localization of claudin-2 with claudin-3, yet another claudin protein, which demonstrated predominant claudin-3 expression among differentiated colonocytes at the crypt top (Figure 1A and 1B). To further confirm, we utilized *in vitro* models of intestinal epithelial cell (IEC) differentiation:

(A) *In vitro* model of spontaneous differentiation: Caco-2 and HT-29 cells, used primarily for IEC differentiation studies, were subjected to spontaneous differentiation as described in the “Methods”. Established markers of IEC differentiation, the Alkaline Phosphatase (AP) activity and P-21/Cip1 expression served as positive controls [31, 32]. The differentiated cell monolayer (at day-21 post-culture) contained well-formed domes and demonstrated significantly higher AP-activity (*versus* D-1 post-culture; p>0.001) (Figure 1C and 1D). Immunoblot analysis using cell lysates collected at different time-points during the differentiation process further demonstrated spontaneous increase in P-21 expression as cells underwent terminal differentiation (D-7 to D-21 post-culture; p>0.001 *versus* D-1 post-culture, Figure 1E and 1G). Overall, these data supported validity of our model. We documented highest claudin-2 expression in least differentiated cells during the initial phase of cell culture which decreased sharply as cells differentiated (p>0.001 at D-7 *versus* D-1 post-culture cells). In contrast, claudin-3 expression increased progressively as cells differentiated [33] while claudin-4 expression remained largely unaltered (Figure 1E). We found similar decrease in claudin-2 expression in HT-29 cells subjected to spontaneous differentiation and a contrasting increase in P-21 expression. Claudin-4 expression remained unaltered (Figure 1H-1J). The qRT-PCR using total RNA isolated from cells grown under similar culture conditions also demonstrated significant decreases in claudin-2 expression and contrasting upregulation of P-21/Cip1 mRNA expression during the differentiation process (Supplementry Figure 1). Overall, these findings supported an inverse association between claudin-2 expression and colonocyte differentiation.

(B) *In vitro* model of pharmacologically induced differentiation: The HDAC inhibitors (HDACi) are potent inducers of growth arrest and terminal differentiation including in colon cancer cells [34, 35]. Therefore, to validate above findings, we cultured Caco-2 cell monolayer in the presence of increasing concentration of sodium butyrate (NaB), a broad range HDACi [34]. P-27/Kip1, a cell cycle inhibitor and differentiation marker, served as positive control. Immunoblot analysis demonstrated, as expected, a significant and dose-dependent increase in P-27/Kip1 expression in Caco-2 cells subjected to NaB-treatment *versus* control cells. In same samples, claudin-2 expression decreased sharply and was lowest in cells exposed to the highest NaB concentration (Figure 2A-2C; P<0.01 *versus* control cells). We found similar decrease in claudin-2 expression and contrasting increase in P-27 expression in Caco-2 cells subjected to TSA (Trichostatin-A; selective inhibitor of class-I and -II HDACs; or SAHA (suberoylanilide hydroxamic acid or *Vorinostat*; a potent and reversible inhibitor of class-I and -II HDACs, currently employed in clinical trials) (p<0.01 and p<0.001 respectively; Figure 2D-2I). We also determined whether HDACi-dependent decrease in claudin-2 expression also associated with altered cellular distribution of claudin-2 protein. Co-immunofluorescence analysis was performed for claudin-2 and ZO-1, yet another TJ-protein. As expected, anti-claudin-2 immunoreactivity in control cells was predominantly membrane localized though trace amounts of the protein was also detected inside the cell. ZO-1 immunoreactivity was detected primarily in the membrane localization. SAHA-treatment suppressed anti-claudin-2 immunoreactivity in all cellular localization without majorly affecting ZO-1 expression and/or cellular distribution (Figure 2J). We found similar changes in claudin-2 expression in HT-29 cells subjected to the HDACi-treatment (Supplementary Figure 2). Overall, these findings confirmed an inverse association between claudin-2 expression and colonic epithelial differentiation, and also implied that the colonic claudin-2 expression is epigenetically regulated.

### Genetic manipulation of claudin-2 expression inversely affects differentiation and growth properties in CRC cells

**Figure 3:**
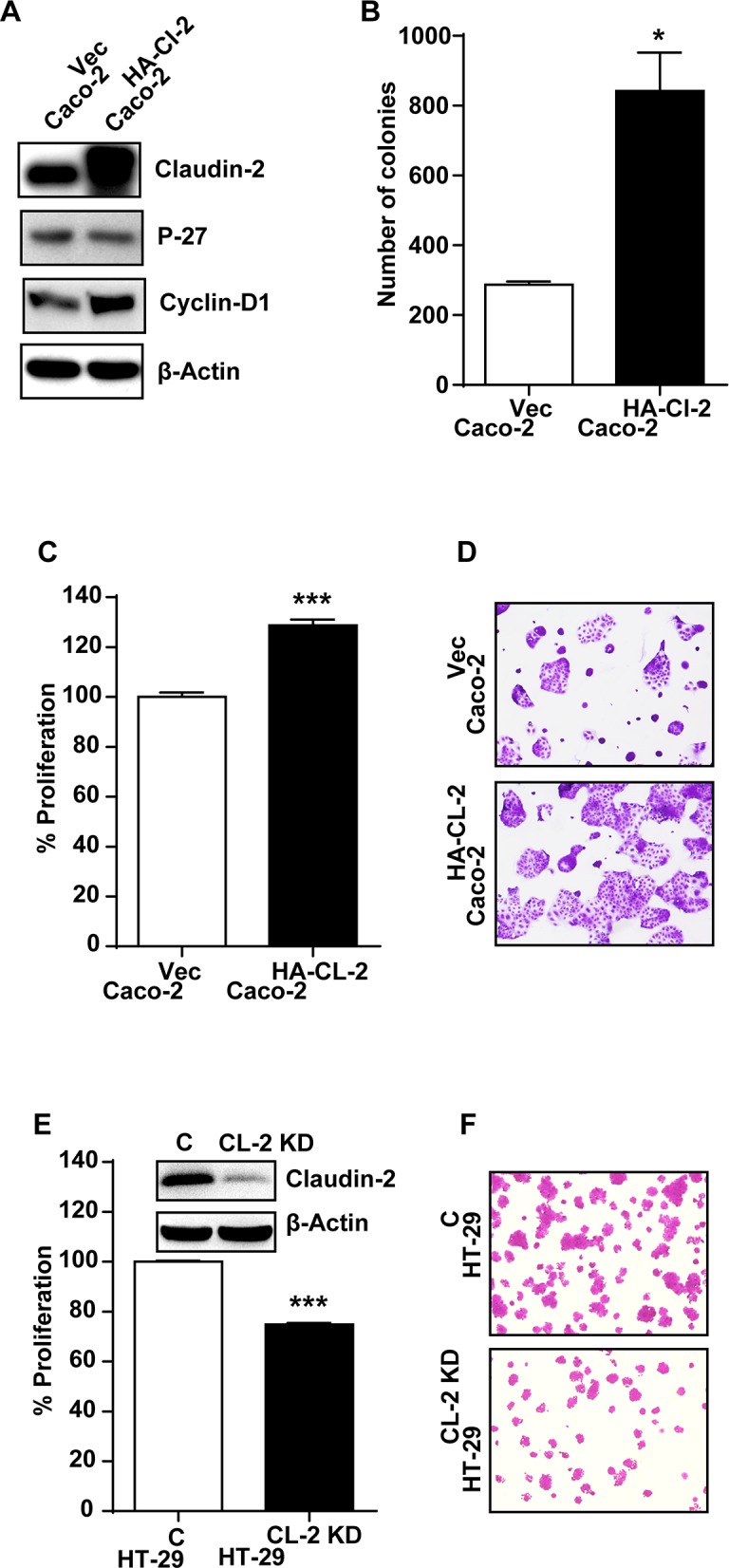
Genetic modulation of claudin-2 expression modulates differentiation program and growth properties in colon cancer cell **(A)** Immunoblot analysis using cell lysate from Caco-2 cells stably overexpressing HA-tagged (c-terminal) claudin-2 cDNA construct (Caco-2^HA-CL-2^) and control cells.; **(B)** Soft agar assay using control and claudin-2 overexpressing Caco-2^HA-CL-2^ cells.; **(C-D)** Crystal violet staining demonstrating increase in cell growth with claudin-2 overexpression (Caco-2^HA-CL-2^).; **(E-F)** Knockdown of claudin-2 in HT29 colon cancer cell (HT29^CL-2-KD^) and control cells representing crystal violet staining and representative calirometric analysis to asses cell growth.; Three independent experiments were done and outcome is presented as mean ± S.E.M. ^*^P<0.05, ^**^P<0.01 and ^***^ P<0.001 versus control.

Based upon above findings, we hypothesized that the upregulated claudin-2 expression in CRC promotes dedifferentiation and proliferative programs to promote colon carcinogenesis. To examine, we utilized Caco-2 cells stably overexpressing HA-tagged and full-length human claudin-2 cDNA (Caco-2^HA-Cl2^ cells) [3]. To complement, we stably inhibited claudin-2 expression in HT-29 (HT-29^CL-2KD^) cells, using anti-human claudin-2 shRNA. Caco-2 cells expressing the empty cloning vector and HT-29 cells expressing control shRNA served as respective controls. Immunoblot analysis confirmed robust claudin-2 expression in Caco-2^HA-Cl2^ cells. A sharp increase in cyclin-D1, a proliferation marker and contrasting decrease in P-27 expression accompanied the increase in claudin-2 expression in Caco-2^HA-Cl2^ (versus control Caco-2 cells; Figure 3A). Further analysis also demonstrated significant growth advantage for Caco-2^HA-Cl2^ cells when grown in the soft-agar based cell culture (colony formation; p<0.05;Figure 3B) or regular culture dishes (p<0.001; Figure 3C and 3D), compared to control cells. In contrast, HT-29^CL-2KD^ cells demonstrated significant decrease in growth compared to the control cells (p<0.001; Figure 3E and 3F). Overall, these data supported causal association between claudin-2 expression and differentiation/proliferative status of the CRC cells.

### HDACi inhibit claudin-2 transcription in differentiating colon cancer cells

**Figure 4:**
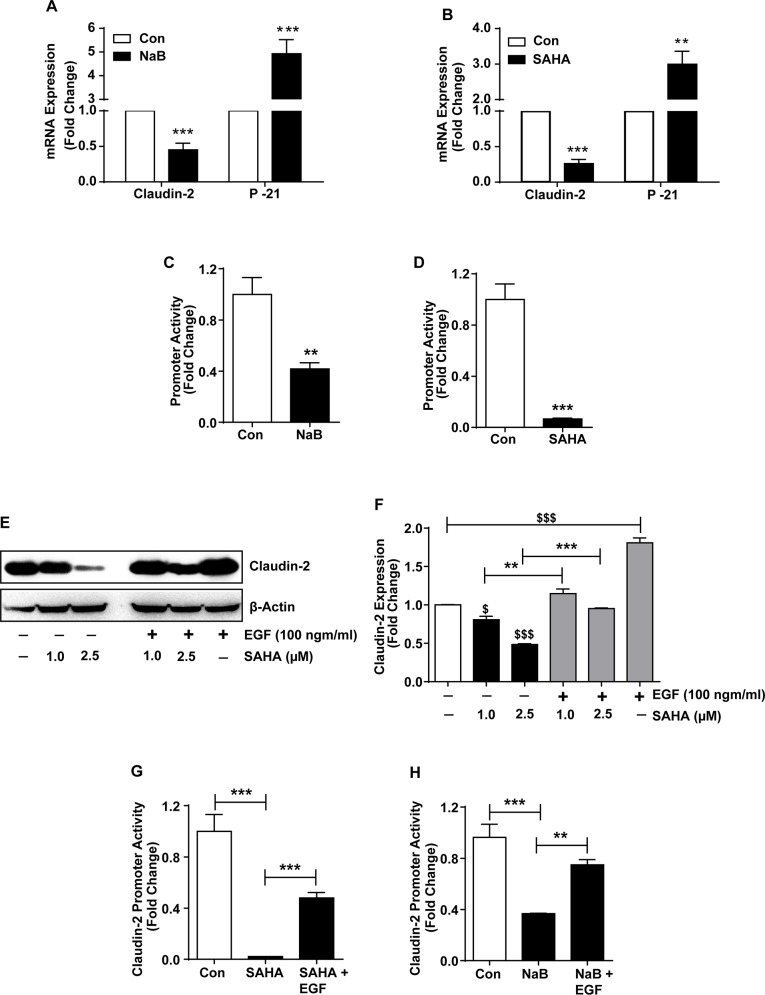
HDAC-inhibitors (HDACi) inhibit claudin-2 transcription to regulate its expression in differentiating colon cancer cells **(A-B)** qRT-PCR of claudin-2 and P-21^waf1/cip1^ using total RNA isolated from SAHA or NaB-treated cells. ; **(C-D)** Claudin-2 promoter activity in control and SAHA or NaB-treated CRC cells.; **(E-F)** Immunoblot and densitometry analysis of claudin-2 in total cell lysate of colon cancer cell subjected to SAHA with/without EGF (100ng/ml). EGF-alone treatment was used as positive control.; **(G-H)** Claudin-2 promoter activities in response to NaB or SAHA treatments. Three independent experiments were done and data is presented as mean ± S.E.M. ^*^P<0.05, ^**^P<0.01 and ^***^ P<0.001 *versus* control.

HDACs are essential components of the co-regulatory complexes and key mediators of gene transcription. However, HDACs also help regulate post-transcriptional regulation of the gene expression [36, 37]. Therefore, we examined if HDACi-dependent claudin-2 regulation is also at transcriptional level. We performed qRT-PCR analysis using total RNA isolated from the control and NaB or SAHA-treated cells. P-21 expression served as positive control. As demonstrated in Figure 4A, claudin-2 mRNA expression was significantly decreased in NaB-treated cells (p>0.001). P-21 mRNA expression (p>0.001) was significantly increased in same samples compared to untreated Caco-2 cells. We found similar decrease in claudin-2 expression (p<0.001) and contrasting increase in P-21 expression (p<0.01) in cells subjected to SAHA-treatment (Figure 4B). However, these outcome from the qRT-PCR analysis could not differentiate if observed effects on claudin-2 mRNA expression was due to altered mRNA transcription or mRNA stability. To clarify, we performed promoter reporter assay using a human claudin-2 promoter luciferase reporter construct (Cldn2pro), as previously described [3]. The Cldn2pro or control reporter constructs along with Renilla were transiently transfected into Caco-2 cells and effect of HDACi-treatment was evaluated. In brief, 12-hours post-transfection of cells with respective promoter reporter construct, cells were exposed to NaB (2.5 mM) or SAHA (2.5μM) treatment. Twenty-four hours post-treatment, samples were collected and luciferase activity was determined. A significant decrease in claudin-2 promoter activity in both, NaB (p>0.01) and SAHA-treated cells (p>0.001) compared to control cells suggested that the HDACi regulate claudin-2 expression by modulating its mRNA transcription during colonic epithelial differentiation (Figure 4C and 4D).

### HADCi regulate claudin-2 expression by modulating EGFR/ERK1/2 signaling

Epidermal growth factor receptor (EGFR), a receptor tyrosine kinase, which promotes cell proliferation and dedifferentiation, is abnormally upregulated in variety of epithelial tumors including CRC [38–40]. Moreover, our laboratory has demonstrated key significance of the EGFR/ERK1/2 signaling in regulating colonic claudin-2 expression and upregulation in CRC cells [3]. HDACi inhibit EGFR-expression and tumorigenic properties in CRC cells [41]. We therefore investigated whether HDACi dependent regulation of claudin-2, in our studies, was dependent on modulation of the EGFR/ERK1/2 signaling. Since, EGFR activation promotes claudin-2 expression in CRC cells, we examined if exogenous EGF will counteract the inhibitory effect of HDACi upon claudin-2 expression. Confluent monolayer of Caco-2 cells were rendered quiescence by serum starvation (overnight) and then subjected to SAHA-treatment with or without the presence of EGF (100ng/ml) in the culture medium. Cell lysates prepared from the cells 24-hours post-treatment were subjected to immunoblot analysis. As expected, EGF-treatment of Caco-2 cells significantly increased claudin-2 expression (p<0.001). Remarkably, EGF-treatment also resulted in significant recovery of the SAHA-dependent decreases in claudin-2 expression (p<0.001; *versus* SAHA alone; (Figure 4E and 4F) suggesting that the HDACi may regulate caudin-2 expression by inhibiting EGFR-expression and dependent signaling. Indeed, we found sharp inhibition of P-ERK1/2 expression in SAHA-treated cells (*versus* control; Figure 6A). To further confirm, we examined if EGF-treatment would also rescue the HDACi-dependent inhibition of Cldn2pro luciferase reporter activity. Caco-2 cells were transfected with control or Cldn2pro reporter constructs. Twenty-four hours post-transfection, cells were sub-cultured into control, SAHA-, SAHA+EGF-groups, subjected to respective treatments and effect on claudin-2 promoter activity was determined. A significant recovery of claudin-2 promoter activity in SAHA+EGF cells compared to SAHA-alone cells (p<0.001) supported the postulation that HDACi regulate claudin-2 transcription in EGFR-dependent manner (Figure 4G). We found similar outcome when cells were subjected to NaB-treatment with or without EGF-treatment (p<0.01 *versus* NaB-treated cells; Figure 4H). Taken together, our data demonstrated that the HDACi regulate claudin-2 expression in differentiating CRC cells by modulating EGFR-signaling.

### HDAC-4 regulates claudin-2 expression in differentiating colon cancer cells

**Figure 5:**
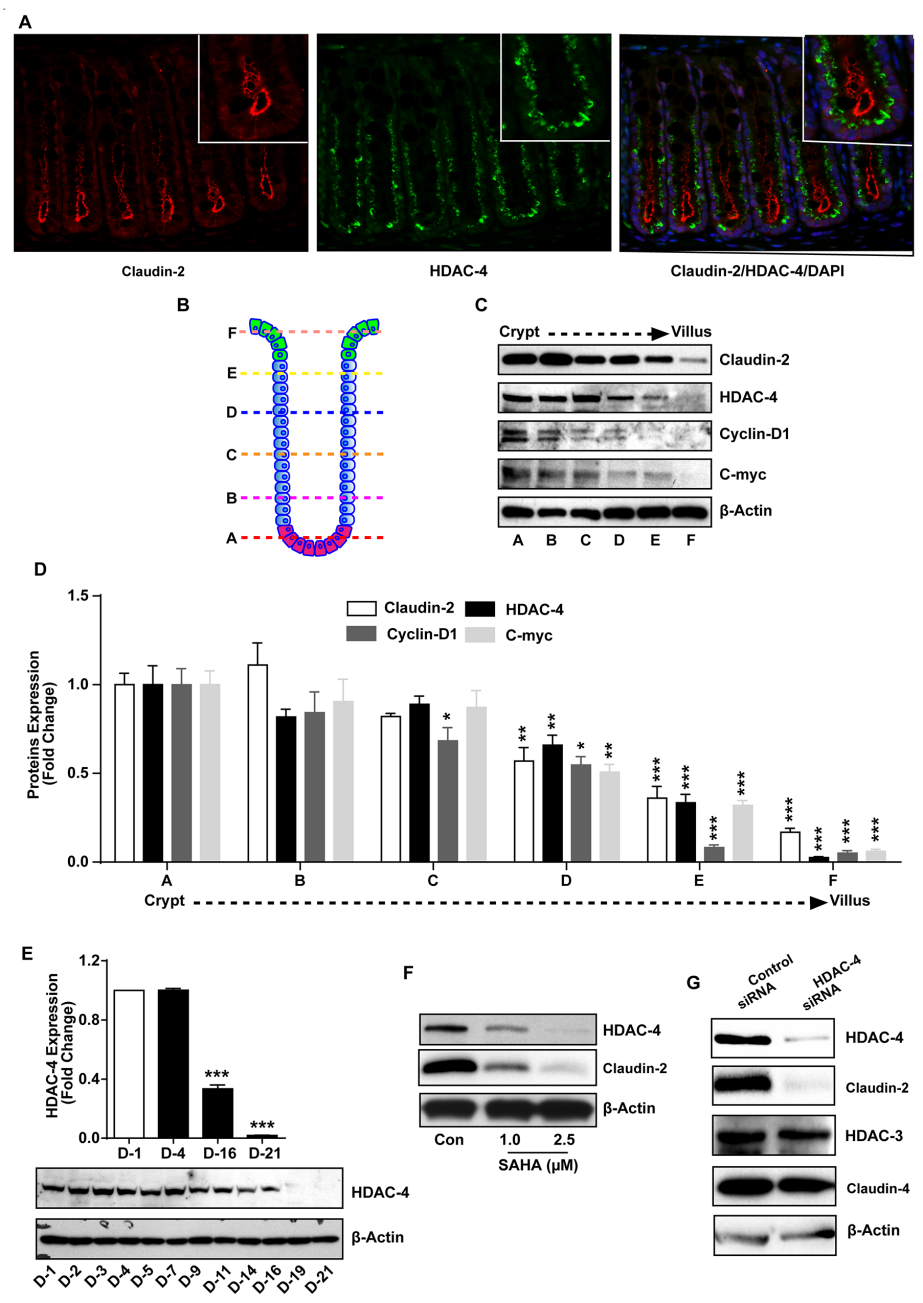
HDAC-4 regulates colonic claudin-2 expression **(A)** Immunofluorescence staining using paraffin sections of normal mouse colon using anti-HDAC-4 (Green) and -claudin-2 (Red) antibody.; **(B)** Cartoon representing different section of crypt which is divided in six portion from base (A) to top (F). Epithelial cells were isolated sequentially form these section using manual fractionation.; **(C-D)** Immunoblot and densitometry analysis of claudin-2, HDAC-4, cyclin-D1 and c-myc using lysate prepared from fractions isolated from colon crypts (form base to top of the crypt).; **(E)** Immunoblot and densitometry analysis using anti-HDAC-4 antibody using lysates prepared from Caco-2 cells undergoing spontaneous differentiation.; **(F)** Immunoblot analysis using lysates prepared from Caco-2 cells subjected to SAHA treatment in dose dependent manner.; **(G)** Immunoblot analysis using cell lysate from control and HDAC-4 knockdown cells. Represent Immunoblot of claudin-2, HDAC-4, HDAC-3 and claudin-4. Three independent experiments were done and represented as mean ± S.E.M. ^*^P<0.05, ^**^P<0.01 and ^***^ P<0.001 *versus* control.

We further examined specific HDAC-protein responsible for regulating colonic claudin-2 expression during epithelial differentiation. In this regard, previous studies have reported colonic HDAC-4 expression pattern to be similar to claudin-2 expression and also its CRC promoting function [42]. To examine, we performed co-immunofluorescence staining for claudin-2 and HDAC-4 using mouse colon, which demonstrated co-existence of both proteins in the colonic crypt in similar location (Figure 5A). To further confirm, we performed fractionations of the colon crypt from crypt bottom to the crypt top, using established protocol described in “Methods” [42]. Immunoblot analysis using these fractions confirmed the outcome from immunostaining and differentiation analyses (Figure 1E), as highest claudin-2 and HDAC-4 expression were detected in fraction-A (the crypt bottom) and least expression in fraction-F (the crypt top). Further analysis demonstrated similar expression pattern for cyclin-D1 and c-Myc, established proliferative markers and Wnt-signaling targets (Figure 5B-5D). Based upon these interesting findings, we determined if HDAC-4 expression also decreases in differentiating Caco-2 cells. Samples from Caco-2 cells subjected to spontaneous differentiation and used for determination of claudin-2 and P-21 expression (Figure 1E) were immunoblotted to determine HDAC-4 expression. As shown in Figure 5E, immunoblot analysis demonstrated a graded however significant decrease in HDAC-4 expression in differentiating cells. We found similar graded decrease in HDAC-4 expression in HT-29 cells subjected to spontaneous differentiation (Supplementary Figure 3). In further studies, we found similar dose-dependent decrease in HDAC-4 expression in Caco-2 cells subjected to SAHA-treatment in correlation with the decrease in claudin-2 expression (Figure 5F). To further determine if observed changes in HDAC-4 and claudin-2 expression were causally associated, we performed genetic inhibition of HDAC-4 expression. Caco-2 cells were transfected with anti-human HDAC-4 or control siRNA. Immunoblot analysis using cell lysate from control or anti-human HDAC-4^siRNA^ expressing cells confirmed sharp inhibition of HDAC-4 expression. In same samples, expression of HDAC-3 remained largely unmodified and thus supported specificity of the gene silencing. Importantly, claudin-2 expression in HDAC-4^siRNA^ cells was also inhibited to its nadir while claudin-4 expression remained largely unaltered (Figure 5G). Taken together, above findings supported causal role of HDAC-4 in regulating claudin-2 expression during colonocyte differentiation, and in CRC cells.

**Figure 6:**
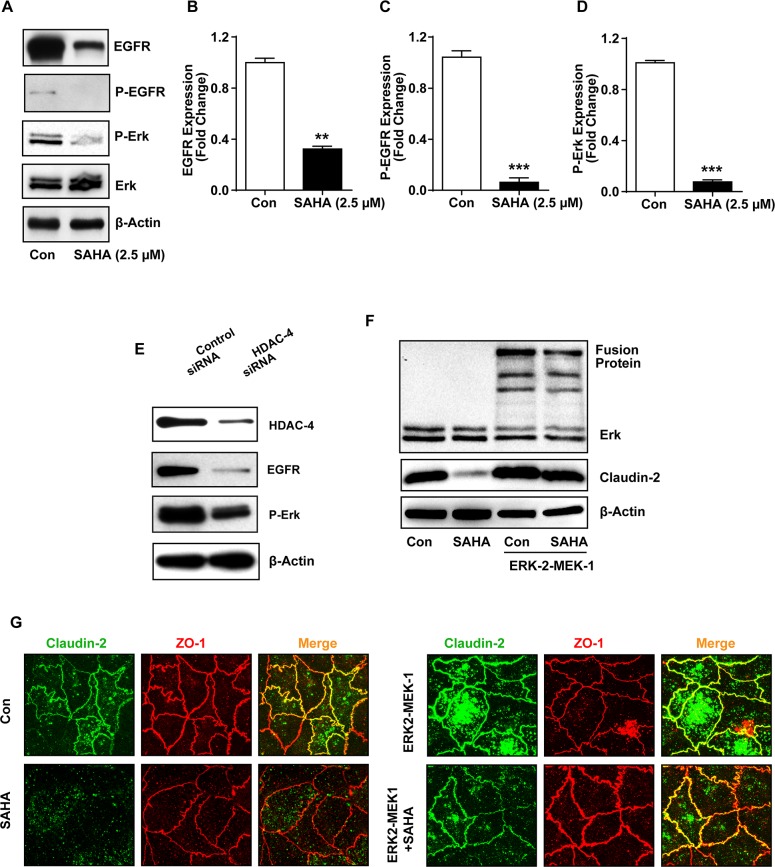
HADC-4 regulates claudin-2 expression in EGFR/ERK1/2-dependent manner **(A-D)** Immunoblot and densitometry analysis of EGFR, P-EGFR, P-ERK1/2 in total cell lysates of colon cancer cell treated with SAHA.; **(E)** Immunoblot analysis of EGFR and P-ERK1/2 using lysates prepared from control and HDAC-4 knockdown cells.; **(F)** Immunoblot analysis of claudin-2 and ERK-2(ERK2-MEK-1) in cells transfected with ERK2-MEK-1 construct and treated with or without SAHA.; **(G)** Representative co-immunoflurence staning of claudin-2 (green) and ZO-1(Red) in Caco-2 cells expressing constitutively active ERK-2 (ERK2-MEK-1 fusion protein) with or without SAHA-treatment. Three independent experiments were done and data is presented as mean ± S.E.M. ^*^P<0.05, ^**^P<0.01 and ^***^ P<0.001 *versus* control.

### MAP kinase ERK1/2 signaling rescues HDACi-mediated suppression of claudin-2 expression in differentiating CRC cells

Our overreaching hypothesis is that the HDACi regulate claudin-2 expression in EGFR-dependent manner. In such case, we should also document changes in EGFR expression/activation. To test, we immunoblotted cell lysates from Caco-2 cells subjected to SAHA-treatment, as above. It was interesting that similar to claudin-2 expression, EGFR expression was significantly downregulated in SAHA-treated cells (p<0.01; *versus* control cells) (Figure 6A). Further determinations revealed similar suppression of P-EGFR (Y1173) and P-ERK1/2 expressions in SAHA-treated cells (p<0.001 for both *versus* control cells) (Figure 6A-6D). Further analysis demonstrated similar significant decreases in EGFR and P-ERK1/2 expressions in Caco-2 cells subjected to siRNA-transfection against HDAC-4 (Figure 6E). Considering the key importance of ERK1/2 signaling in EGFR-dependent cellular functions and published data that EGFR/ERK1/2 signaling is critical for maintaining colonic claudin-2 expression, we further determined if HDACi-dependent decrease in claudin-2 expression can be rescued by simply activating the ERK1/2-signaling. To test, we used a constitutive ERK-2/MEK-1 construct. Construction and characterization of this construct has been described before [43]. Cells were transfected with control or ERK-2/MEK-1 construct and 24-hours post-transfection cells were subcultured into control and experimental wells, and subjected to vehicle or SAHA-treatment, respectively. Immunoblot analysis using lysates prepared from these cells confirmed overexpression of ERK-2/MEK-1 construct and a significant recovery of SAHA-induced suppression in claudin-2 expression. Immunoflourescent localization further confirmed this recovery and demonstrated re-expression of claudin-2 protein in the expected cellular localization (Figure 6F and 6G). Taken together, our data demonstrated role of the HDAC4/EGFR/ERK1/2 signaling in regulating claudin-2 expression in differentiating colon cancer cells.

### HDAC4/ERK1/2/Claudin-2 axis is highly upregulated in colon cancer

**Figure 7:**
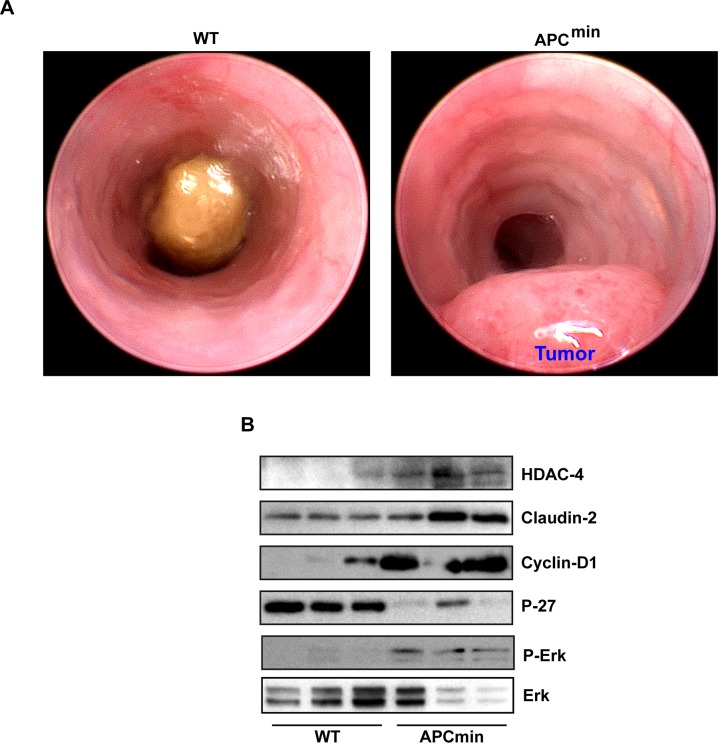
HDAC4/ERK1/2/Claudin-2 axis is highly upregulated in colon cancer **(A)**. Represantative colonoscopic images of APC^min^ mice with wild type littermate confirming colon tumor growth in APC^min^ mice.; **(B)** immunoblot analysis of HDAC-4, claudin-2, cyclin-D1, P-ERK1/2 and P-27^Kip1^ using lysates prepared from wild type mice and APC^min^ mice of the same age and sex.

**Figure 8:**
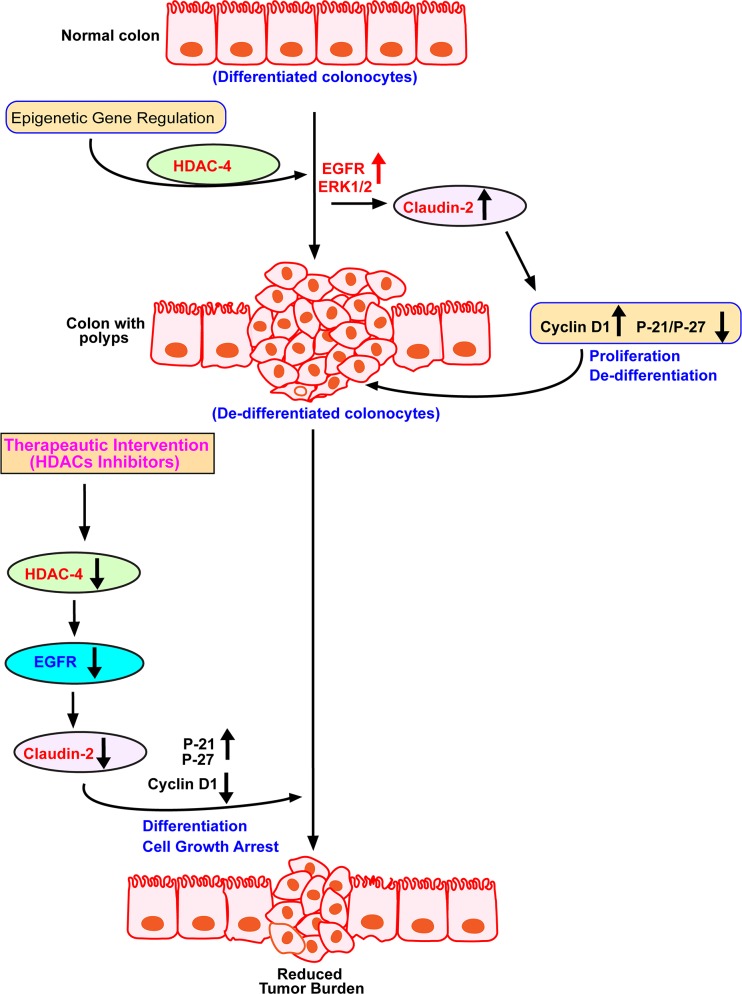
Schematic illustration of epigenetic claudin-2 regulation and therapeutic intervention by HDACs inhibitors for reducing tumor burden by downregulating claudin-2 expression

Considering the pro-tumorigenic role of claudin-2 in colon cancer, we then determined clinical significance of our findings. In this regard, we used APC^min^ mice, the commonly used mouse model of spontaneous intestinal tumorigenesis [44]. APC^min^ mice (8-10 weeks old) were first tested for their colonic tumor burden using mouse colonoscopy (Figure 7A) as colon tumorigenesis in these mice is not as prevalent as intestinal tumor growth [45]. Only those mice that demonstrated colon adenoma/s were sacrificed along with age and sex-matched WT mice (C57/Bl6 strain). Immunoblot analysis was performed using total tissue lysate from the WT-mice and APC^min^ mice colons. Results demonstrated a sharp increase in claudin-2 expression in APC^min^ mice (*versus* WT mice). Further analysis demonstrated similar increases in HDAC-4 and P-ERK1/2 expressions in APC^min^ mice (*versus* WT mice). We also found a sharp increase in Cyclin-D1 expression however contrasting decrease in P-27 expression in APC^min^ mice lysate *versus* WT-mice (Figure 7B). Taken together, our data supported a pro-tumorigenic role of the HDAC4/ERK1/2/Claudin-2 axis potentially by modulating differentiation and proliferation abilities of transformed colonic epithelium (Figure 8).

## DISCUSSION

An appreciation of the colonic crypt organization has key significance in understanding of the processes of colonic epithelial repair/regeneration and/or colon tumorigenesis. In this regard, colonic crypts house the undifferentiated and proliferative colonocytes at the crypt bottom which progressively differentiate as they migrate towards the crypt top [46]. Thus, for proliferative advantages, as in epithelial regeneration and/or colon tumorigenesis, differentiating ability of the colonocytes is compromised. In accordance, studies have demonstrated how mutations in intestinal stem cells expand within the epithelium to generate CRC [47, 48]. Thus, improved understanding of the molecular processes regulating colonocyte differentiation can help unravel novel therapeutic opportunities against colon cancer. In current study, we provide data supporting causal significance of claudin-2 expression in regulating epithelial differentiation among colonocytes and colon cancer cells. Considering significant increases in claudin-2 expression in inflammatory bowel disease (IBD) and colon tumorigenesis [3], these findings can be harnessed for therapeutic advantage.

Claudins are the main regulatory molecules responsible for controlling paracellular permeability via the tight junctions [49]. In this context, claudin-2 status is unique among claudin proteins as its expression is restricted to the leaky epithelia *in vivo*. Moreover, claudin-2 overexpression in cultured cells or *in vivo* increases paracellular permeability for ions and solutes [3, 10]. The outcome from recent reports have further validated biological significance of claudin-2-dependent alteration in the paracellular permeability as genetic loss of claudin-2 expression in mice resulted in insufficiencies in renal Na+ and energy homeostasis, susceptibility to gall bladder stones and nutrient malabsorption (albeit when claudin-15 expression is also inhibited) [50–52]. Remarkably, an increase in the paracellular permeability also characterize the transformed epithelia [53]. Accordingly, significant elevation in claudin-2 expression has been reported in colorectal [3], hepatocellular [54], and gastric cancers [15]. However, emerging studies have also demonstrated non-canonical role of claudin-2 in regulating cellular functions such as cell proliferation, migration and differentiation [3, 14, 54–56]. Indeed, genetic manipulations leading to increased claudin-2 expression in CRC cells also induced cellular proliferation [3, 57]. These findings are well in sync with our current data that claudin-2 expressing cells in the colonic crypt were also Ki-67 positive. Our data using manual fractionation of the colonic crypt, from crypt bottom to crypt top, further support this postulation as expression pattern for claudin-2 strongly matched with the expression for Cyclin-D1 and C-Myc, proliferation markers and Wnt-signaling targets [58]. Similar findings from high throughput transcriptome analysis using colonic crypt fractionation support these findings [59]. Moreover, claudin-2 overexpression in mouse colonic epithelium increased paracellular permeability and colonocyte proliferation [10]. An intricate interplay exist between cell cycle progression and differentiation as cells undergoing differentiation undergo cell cycle arrest. Our data that claudin-2 expression decreases sharply in differentiating colonocytes and/or CRC cells, and claudin-2 expression positively affects cell growth while inhibiting P-21 expression suggests that the increased claudin-2 expression in CRC cells may help promote colon tumorigenesis by facilitating dedifferentiation and cell cycle progression. Our current studies are aimed at deciphering molecular details of these cellular phenomenon in causal association with claudin-2 expression.

Histone hyperacetylation is frequently reported in various cancers and histone deacetylation is reported to induce better prognosis in cancer patients [60]. HDACs are often overexpressed in cancers including CRC and lead to silencing of tumor suppressor genes, which is why HDACi of varied biological capacities are currently in clinical trials [29, 35, 61, 62]. In this regard, HDACi induce cell cycle arrest in CRC cells, and HDACi-mediated growth arrest consistently involves induction of p21 and/or P27 expression [35, 63]. Consistently, we have found that all HDACi tested in our studies inhibit claudin-2 expression while increasing P-21 or P-27 expression. We found similar contrasting relation between claudin-2 and P-21 expression in Caco-2 or HT-29 cells undergoing spontaneous differentiation. Moreover, genetic alteration in claudin-2 expression was sufficient to modulate cyclin-D1 and P-21 expressions in colon cancer cells and modulate growth properties. Similarly, knockdown of claudin-2 by siRNA in lung cancer cells A549 was recently reported to decrease proliferation concomitant with decreases in cyclin-D1 and E1 expression, cell cycle progression factors [64].

Several studies have demonstrated increased expression of the class-I HDACs: HDAC1, HDAC2, and HDAC3, in multiple human cancers, including colon cancer [35, 42, 61]. In contrast, comparatively little is known about the role that class-II HDACs play in colonic epithelial homeostasis and colon tumorigenesis. In current studies, we have identified the role of class-II HDAC, HDAC-4 in regulating claudin-2 expression in causal association with differentiation in colon cancer cells. Our findings are strengthened by the previous report that HDAC-4 is maximally expressed in the proliferative compartment in human and mouse colon [42]. HDAC-4 expression is also upregulated in other cancer types including breast and esophageal cancer [65, 66]. We have documented maximal claudin-2 and HDAC-4 expression in the proliferative compartment in mouse colonic epithelium and colon tumors in APC^min^ mice. We have documented similar association between HDAC-4 and claudin-2 expression in colon cancer cells undergoing spontaneous or HDACi-induced differentiation. Moreover, siRNA depletion of HDAC-4 or overexpression of claudin-2, in Caco-2 cells, had opposite effect on P-21 expression. A specific role of HDAC-4 in upregulating P-21 expression in CRC cells by promoter regulation is reported [42]. Of interest, siRNA-depletion of HDAC-4 expression in colon cancer cells has been reported not to affect expression of other HDACs. Also in our studies, siRNA depletion of HDAC-4 did not affect HDAC-3 expression suggesting the effects of HDAC-4 on claudin-2 expression and differentiation/proliferative properties of CRC cells is exclusive. In future studies, we aim to investigate clinical significance of the association between HDAC-4 and claudin-2 expression in CRC progression and therapy resistance.

Apart from HDACs, EGFR, a receptor tyrosine kinase which promotes cell proliferation and survival, is also expressed abnormally in the tumors of epithelial origin, including CRC. A critical role of EGFR-signaling in colonic epithelial differentiation and stem cell niche is also documented [67]. Studies have further reported causal role of the HDACi in regulating EGFR expression (mRNA transcription) and associated signaling using colon cancer cells [41]. Among claudin proteins, claudin-2 is the most regulated family member as its expression is regulated by the EGFR, C-Met, Ras and PI-3 Kinase signaling [3, 64, 68]. We were the first to demonstrate the key role of EGFR-activation in regulating colonic claudin-2 expression as claudin-2 expression was specifically downregulated in *Waved-2* mice, which are defective in EGFR-tyrosine kinase signaling [3]. In additional studies, we identified specific role of the EGFR/ERK1/2 signaling in promoting claudin-2 expression in colon cancer cells [3]. Thus, our current findings that HDACi-regulate claudin-2 expression by modulating EGFR/ERK1/2 signaling is well in accordance with previous reports. Importantly, we not only found chronically suppressed EGFR expression and EGFR/ERK1/2 signaling in cells treated with SAHA but also upon siRNA depletion of HDAC-4. Our data that constitutive expression of ERK1/2 signaling was sufficient to alleviate HDACi-induced decreases in claudin-2 expression strongly support the causal role of the HADC4/EGFR/ERK1/2 signaling in regulating claudin-2 expression in differentiating colonocytes and in colon tumorigenesis. Our findings however differ from previous report, which demonstrated the role of the class-I HDACs in HDACi-dependent regulation of EGFR expression [41]. Togther, these findings tend to suggest a common role for EGFR expression in HDACs dependent regulation of the cellular phenotype. Importantly, recent studies have emphasized specific role of the deregulated barrier properties in colon adenomas in progression to adenocarcinoma by promoting microbe infiltration and tumor supportive inflammatory signaling [53]. Claudin-2 expression increases paracellular permeability and is highly upregulated in intestinal pathologies associated with leaky gut [3, 69].

Overall, we here demonstrtae a role for HDAC-4 in regulating differentiation and growth properties of colon cancer cells in causal association with EGFR/ERK1/2-signaling and claudin-2 expression. These findings have important implications for combinatorial colon cancer therapy, because HDAC inhibitors undergoing clinical trial in stage IV colon cancer patients have demonstrated class specificity. Furthermore, colon cancer can be receptive or refractory to the anti-EGFR therapy based upon the status of K-Ras mutation [70]. It will be interesting to determine the role of HDAC4/EGFR/ERK1/2 signaling in colon cancer expressing wild type K-Ras versus mutant K-Ras, in association with cancer progression and therapy resistance, in future studies. Our demonstration of an important role for HDAC-4 and claudin-2 in colon cell growth would suggest that inhibitors of class-I and -II HDACs are likely to be more efficient at inhibiting colon cancer cell growth than class I-specific inhibitors. In conclusion, our findings identify claudin-2 as an important regulator of differentiation and growth properties of colonocytes and colon cancer cells. The co-localization of HDAC-4 and claudin-2 in the proliferative zone of the colonic epithelium, in association with EGFR/ERK1/2 signaling is consistent with its physiological role in maintaining cell proliferation and inhibiting maturation.

## MATERIALS AND METHODS

### Antibodies and reagents

The antibodies against claudin-2 and -4 were from Invitrogen (San Francisco, CA, USA), while anti-HDAC-4 antibodies and NaB were purchased from Upstate Biotechnology (NY, USA). The anti-actin antibody, Trichostastin A, and SAHA were from Sigma-Aldrich (St Louis, MO, USA). HDAC-4 siRNA were purchased from Cell Signaling (Beverly, MA USA). Details of these reagents are provided in Supplementary Tables 1 and 2.

### Cell culture and transfection

Caco-2 and HT29 cell lines obtained from the American Type Culture Collection (ATCC). These cells are the most widely used cell model relating with spontaneous or chemical-induced colonic epithelial differentiation [71, 72]. All cells were maintained in media and growth conditions (5% CO2 - 95% air in humified incubator at 37°C). Growth media was supplemented with 10% FBS, 100 U/ml penicillin and 100 μg/treptomycin. Transient transfection experiments were carried out using log phase Caco-2 cell. Briefly, 1×10^5^ Caco-2 cells allowed entering log phase by maintaining them overnight in complete growth media. 2 μg endotoxin free plasmid preparation was mixed with 4.0 μl DNA transfection reagent (Jet Prime; Polyplus-transfection, NY, USA) and volume was made up to 200 μl by tranfection buffer. Reaction mixture was vortex and incubated at room temperature for 10 minutes, thereafter reaction mixture was overlaid on cultured Caco-2 cells followed by addition of 2 ml complete DMEM-culture medium.

### Western blot analysis

The colon cancer cells were lysed in radio-immunoprecipitation assay (RIPA) buffer containing protease and phosphatase inhibitors (1mM phenylmethylsulfonyl fluoride, 10mg/ml aprotinin, and 10mg/ml leupeptin, 10μM sodium orthovanadate). After sonication, lysates were centrifuged at 13,000xg at 4°C for 10 min, protein concentration was determined using Bradford method. After blocking, membranes were incubated at 4°C overnight with the respective antibodies. Blots were incubated for 1 h at room temperature with HRP-conjugated secondary antibody and signal was visualized with horseradish peroxidase-conjugated secondary antibodies using enhanced chemiluminescence (Amersham Biosciences, Piscataway, NJ, USA).

### Claudin-2 promoter activity

Cells were plated one day before transfection. At 40-60% confluence, cells were transfected with pGL3-claudin-2 (Cldn2Pro) and pGL3-control along with Renilla plasmid construct using the Jet Prime transfection reagent, cells were harvested after 48 hours and luciferase activity was measured as described previously using dual-luciferase reporter assay (Promega, Madison, WI, USA) and Synergy-2 Luminometer (BioTechSynergy, Winooski, VT, USA). Transfection efficiency was normalized to *Renilla* luciferase activity of the phRL-TK (Promega, Madison, WI, USA). Fold induction was calculated by dividing the normalized luciferase activity by the value obtained from the control transfection.

### Methods of inducing epithelial differentiation

Caco-2 cells undergo, in culture, a process of spontaneous differentiation that leads to the formation, after two to three weeks, of a monolayer of polarized cell, coupled by tight junctions and expressing several morphological and functional features of intestinal epithelial cell differentiation. Accordingly, 1×10^6^ cells were plated in 35 mm plastic culture dishes and culture medium was changed every second day. Cells were harvested at times indicated and subjected to Western-blot analysis. To induce forced differentiation in these cells using pharmacological inducers known as histone deacetylase (HDACs) inhibitors, Trichostatin A (TSA), sodium butyrate (NaB) and Suberoyanilide hydroxamic acid (SAHA) were used as indicated concentrations. Of note, these HDACs inhibitors are known to induce forced epithelial differentiation among colon cancer cells.

### RNA isolation and qRT-PCR analysis

RNA was extracted from samples using the RNAsy mRNA Purification Kit (Qiagen, Valencia, CA, USA). Reverse-transcription was performed using iScript cDNA synthesis kit (Bio-Rad, Hercules, California, CA, USA). The real-time PCR reactions were performed with appropriate primers (Supplementary Table 3) using 25 ng of cDNA/reaction and 2×iQTM SYBR Green Supermix (Bio-Rad, Hercules, CA, USA).

### Alkaline phosphatase activity assay for cell differentiation

Alkaline phosphatase (ALP) activity was measured to determine the differentiation of enterocytes during spontaneous and forced induced differentiation by HDACs inhibitors. Briefly, cells were homogenized in lysis buffer. Alkaline phosphatase activity, marker of IEC differentiation, was measured in whole cell lysates as described previously using p-nitrophenyl-phosphate (sigma, St. Louis, MO, USA) as subtract. Results were expressed as fold change of ALP activity in treated cells compared to control. The total protein concentration was determined by Bradford protein assay (Bio-Rad, Hercules, CA, USA).

### Anchorage-independent growth assay

The anchorage-independent growth assay was conducted as described previously [4]. In brief 10,000 cells was plated in 1 ml of DMEM with 0.6% low-melting agarose and 10% FBS and overlaid onto 1 ml of DMEM with 0.6% agarose and 10% FBS in six well plate. After 2 weeks, plates were photographed and colonies were counted by using the GelCount (Oxford Optronix, SciProInc, NY, USA) colony-counting machine.

### Cell proliferation assay

To study the causal association of claudin-2 in proliferation, claudin-2 manipulated cells were plated in 12-well cell culture plates (30000 cells/well) for 3 days, then fixed chilled methanol for 15 minutes. The fixed cells stained with 0.5% Crystal Violet for 5 minutes at room temperature before being emptied, washed with distilled water, and air-dried. Representative images were taken using Nikon eclipse microscope. Bound dye was solubilized in 2% sodium deoxycholate for 30 minutes at 37°C and absorbance was measured in each aliquot of each well at 630 nm using a Synergy (Bio-teck, Winooski, VT, USA) microplate reader.

### Mouse colonic crypt to villus fractions and colonoscopy

The sequential isolation of mouse colonic epithelial cells fractions along the crypt to villus was conducted as described previously [59, 73]. In brief, colon was chopped into 1cm pieces and incubated with 2 mm EDTA in PBS for 10 min and vigorously agitated before collecting supernatant and then repeated similar steps to get sequential crypt to villus fractions. To detect presence of colon tumors, APC^min^ mice and WT littermates were followed up for tumor detection using colonoscopy as described previously using high resolution mice colonoscope from Karl Stroz, (Tuttlingen, Germany) [74]. All animal studies were done using Institutional approved practices and animal protocol.

### Statistical analysis

Data were analyzed with Graph Pad Prism Version 6.0 software and are presented as mean ± SEM. Statistical analysis was performed by Student’s t test, 1-way ANOVA analysis of variance. P value of less than 0.05 was considered significant.

### Financial support

This work was supported by BX002086 (P.D.), DK088902 and BX002761 (A.B.S.).

## SUPPLEMENTARY MATERIALS FIGURES AND TABLES


