# Supplementary material for: HDAC-4 regulates claudin-2 expression in EGFR-ERK1/2 dependent manner to regulate colonic epithelial cell differentiation
Source: Oncotarget. 2017 Sep 23;8(50):87718–36. doi: 10.18632/oncotarget.21190 (PMC5675667; doi:10.18632/oncotarget.21190)
Supplement: Supplementary file 1 [file oncotarget-08-87718-s001.pdf]

HDAC-4 regulates claudin-2 expression in EGFR-ERK1/2 dependent manner to regulate colonic epithelial cell differentiation

SUPPLEMENTARY MATERIALS

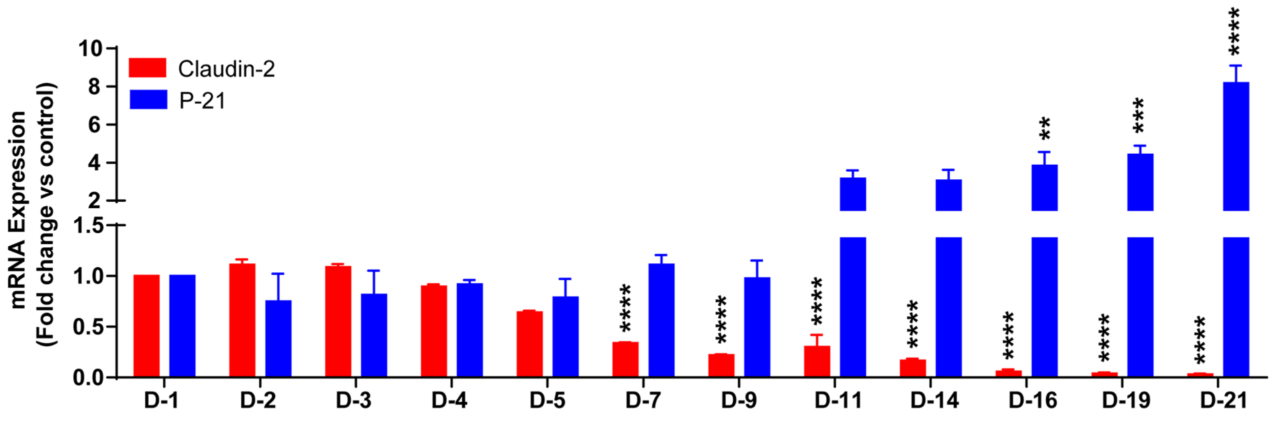

Supplementary Figure 1: qRT-PCR analysis of claudin-2 and P-21$^{waf1/cip1}$ in Caco-2 cell model of spontaneous differentiation. AT least three independent experiments were performed. Data is presented as mean ± S.E.M. **P<0.01 and *** P<0.001 *versus* control.

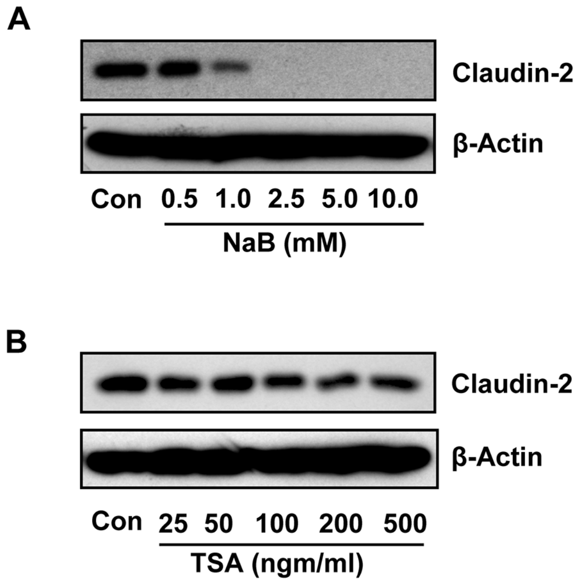

Supplementary Figure 2: (A-B). Immunoblot analysis using anti-claudin-2 antibody and cell lysate prepared from HT29 cells treated with NaB and TSA.

Supplementary Table 1: Reagents and antibody

SN#	Antibody	Company	Catalog#
1	Claudin-2	Invitrogen San Francisco, CA, U.S.A.	325600
2	Claudin-4	Invitrogen San Francisco, CA, U.S.A.	329400
3	β-Actin	Sigma, St Louis, MO, U.S.A.	A2228-100UL
4	HDAC-4	Upstate NY, U.S.A.	ABE262
5	HDAC-3	Upstate NY, U.S.A.	05813
6	ZO-1	Invitrogen San Francisco, CA, U.S.A.	617300
7	P-27	Santa Cruz biotechnology Dallas, U.S.A.	Sc-528
8	p-21	Santa Cruz biotechnology Dallas, U.S.A.	Sc-6246
9	EGFR	Invitrogen San Francisco, CA, U.S.A.	MAS-13269
10	P-EGFR	Santa Cruz biotechnology Dallas, U.S.A.	Sc-101668
11	ERK	Cell Signaling Beverly, MA, U.S.A.	9102s
12	P-ERK	Cell Signaling Beverly, MA, U.S.A.	4370s
13	Cyclin-D1	Cell Signaling Beverly, MA, U.S.A.	2978s
14	C-myc	Santa Cruz biotechnology Dallas, U.S.A.	Sc-40
15	HDAC-4 siRNA	Cell Signaling Beverly, MA, U.S.A.	7595

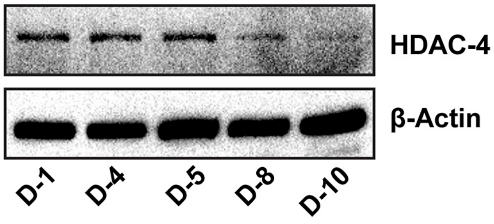

Supplementary Figure 3: Immunoblot analysis using anti-HDAC-4 antibody and cell lysate from HT29 cells subjected to spontaneous differentiation.

Supplementary Table 2: Inhibitors

SN#	Inhibitor	Company	Catalog#
1	Sodium Butyrate	Millipore Billerica Massachusetts, U.S.A.	567430
2	Trichostatin A	Millipore Billerica Massachusetts, U.S.A.	647925
3	Suberoylanilide hydroxamic acid	Sigma St Louis, MO, USA	SML0061

Supplementary Table 3: Primers sequence

SN#	Primer	Sequence
1	Claudin-2(Forward)	ctccctcgcctgcattatctc
2	Claudin-2(Reverse)	cagtggtgagaagtcccg
3	P-21 (Forward)	tgtccgtcagaacccatgc
4	P-21(Reverse)	aaagtcgaagttccatcgctc
